# Emerging Applications for Computer Vision and Artificial Intelligence in Management of the Cardiovascular Patient

**DOI:** 10.14797/mdcvj.1263

**Published:** 2023-08-01

**Authors:** Peter Osztrogonacz, Ponraj Chinnadurai, Alan B. Lumsden

**Affiliations:** 1 Methodist DeBakey Heart & Vascular Center, Houston Methodist, Houston, Texas, US; 2 Vascular and Endovascular Surgery, Semmelweis University, Budapest, Hungary; 3 Occam Labs, London, UK

**Keywords:** artificial intelligence, AI, telenursing, telemedicine, mental workload

## Abstract

Artificial intelligence and telemedicine promise to reshape patient care to an unprecedented extent, leading to a safer and more sustainable work environment and improved patient care. In this article, we summarize how these emerging technologies can be used in the care of cardiovascular patients in such ways as fall detection and prevention, virtual nursing, remote case support, automation of instrument counts in the operating room, and efficiency optimization in the cardiovascular suite.

## Introduction

In Issue 19.2, the article “Cardiovascular Nursing Workforce Challenges: Transforming the Model of Care for the Future” described the development and initiation of virtual intensive care for the cardiovascular patient.^1^ It outlined not only how remote monitoring can be successfully implemented in the intensive care unit (ICU), but also its promise of developing predictive algorithms based on the huge amount of data gathered during the process.

Currently, the lack of human resources (especially registered nurses) represents one of the most pressing matters in health care in the United States (US), and many fear the detrimental effect and long-term implications. Consequently, efforts have been made to prevent employees leaving the field, including salary increases, introduction of contract labor, and independent site staffing. However, these attempts have not appropriately addressed the overall challenge departments face with staffing to care for those with cardiovascular diseases.^1^ What could be the reason behind that?

Patients with cardiovascular morbidities represent one of the most frail patient populations, and their acuity is taxing on nursing and medical staff alike. Simply put, providing care for relatively healthy and young patients may be easier and more rewarding, causing human resource migration to fields with those patients. At the same time, the increase in life expectancy—a trend observed prior to the COVID-19 pandemic—has led to an expanding population suffering from cardiovascular diseases.^2^ Consequently, we must address the conundrum of finding a solution to the lack of sufficient human resources in order to provide adequate care for this growing and frail patient population. The answer may lie in embracing new technologies and adopting them according to unmet clinical needs.

To glimpse possible future applications, we summarize how artificial intelligence (AI) and telemedicine can be used to address some of the current challenges encountered in the care of cardiovascular patients: fall detection and prevention, virtual nursing, remote case support, automation of instrument counts in the operating room, and efficiency optimization in the cardiovascular suite. These approaches and technologies represent the tip of a tsunami of innovation based upon video analysis, data capture, and the development of predictive algorithms that eventually will transform the diagnostic and therapeutic process in cardiovascular patients. These innovations in health care cover a broad spectrum, from using easily available technologies (eg, headsets during operations for better communication among team members) to advanced application of AI that helps employees perform monotonous, repetitive tasks.

## Fall Detection and Prevention

According to the World Health Organization (WHO), falls are the second most common cause of unintentional injury and death in the world, and fatal falls are the most common among adults over 60 years.^3^ Additionally, fall-related death in the US doubled over the 10-year period from 2010 to 2020.^4^ Nationwide, $50 billion is spent annually on nonfatal falls, with fatal falls in those over 65 costing $754 million.^5^ As the world’s population ages,^6^ the incidence and cost of falls will likely increase unless the medical and biomedical community join forces to mitigate this risk. Various reports on fall detection and prevention systems are based on wearables or non-wearables. Wearable-based systems usually have a sensor either on the waist, wrist, or spine and use accelerometer, gyroscope, magnetometer, inertial measurement unit, or surface electromyography to detect the fall.^7,8^ Additionally, assistive walking devices can be used to prevent a fall, as demonstrated in a simulated environment by Kumar et al.^9^ However, lack of compliance may prevent widespread utilization of wearable systems for fall detection in the elderly.

In contrast, non-wearable fall detection and prevention systems do not require devices worn by patients. Fall detection can be carried out either via video surveillance or using floor-based sensors. In the former, captured images are analyzed by different algorithms to ensure fall detection. Floor-based sensors (ground reaction force and pressure sensors) monitor falls by analyzing the force exerted by the feet. The implementation of such technologies allows for the identification of falls in a timelier manner and enables expeditious treatment. Non-wearable fall prediction has been implemented at Houston Methodist Hospital based on the work of Wang et al.^10^ Machine learning made it possible to identify those patients at high risk for a fatal fall and is capable of doing so based on limited information. As a result, the medical staff gains information on fall risk at the time of admission. Increased risk for fall is taken into account while providing care for these patients in terms of more frequent and closer observation. Furthermore, fall prevention measures can be implemented by installing load cells under the sickbed,^11^ which give information about postural changes, including getting out of bed, for the medical staff. Additionally, video monitoring is capable of detecting bed-exits by patients, contributing to a lower risk environment for fall risk.^12,13^ Video monitoring complemented with open-programming software can be used to draw lines around patients, which serve as boundaries and alert the medical staff if the patient breaches the predefined borders.^14^

Fall detection and fall prevention systems offer an invaluable protection net for patients at risk of a fall in a hospital setting. Therefore, we urge every healthcare institution to implement such a safety measure to provide the safest environment for their patients.

## Virtual Nursing

The WHO estimates that nurses and midwifes account for 50% of the global healthcare workforce,^15^ providing the backbone of human resources in medicine. Still, according to the Health Workforce Projection of Health Resources & Service Administration, a nationwide shortage of approximately 78,610 full-time equivalent registered nurses is predicted by 2025.^16^ Efforts have been made to increase the nurse workforce, including hiring bonuses, salary increases, introduction of contract labor, and independent site staffing. However, these attempts have fallen short in addressing the workforce shortage.

**Figure 1:**
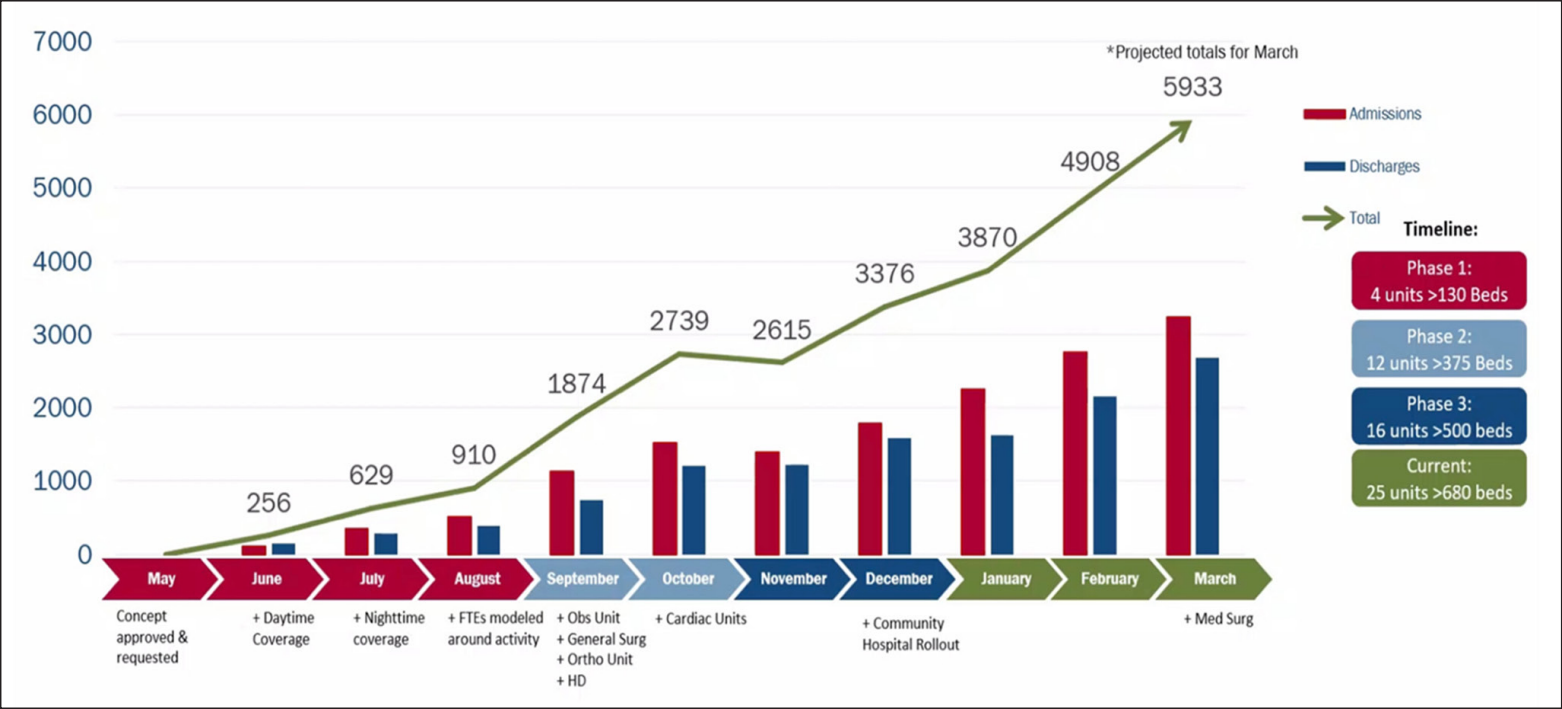
Telenursing volumes at Houston Methodist facilities 2022–2023. Within less than a year, we achieved a 20-fold increase in telenursing volume.

Telenursing provides a possible solution for staffing-related shortages by reducing bedside workload. At Houston Methodist Hospital, we have implemented telenursing with great success. Currently 25 units with more than 680 beds utilize this innovative approach (Figure 1). Instead of introducing new technology, we confine ourselves to using existing systems such as tablets, audiovisual systems, and secure messaging. Off-site nurses are on call to interact with patients via tablets and to complete admission questionnaires, coordinate with on-site staff, and review discharge instructions with the patients. This approach leads to improved capacity management by enabling the bedside team to focus more on in-person tasks while the telenursing team uses their credential-level skills for high-degree patient interaction. Ultimately, it results in a better patient experience due to one-on-one dedicated and personalized interaction with a nurse, uninterrupted discussions, and a more predictable discharge timeline.

## Remote Case Support

The COVID-19 pandemic showed the importance and value of remote case support in the operating room. Virtual connection makes expert proctors more accessible for a wider array of hospitals while allowing the proctor to work from home. The key elements for remote case support include a low latency reliable internet connection, encrypted data transfer, and bidirectional audiovisual connection. Additionally, the proctor must be able to navigate the camera and to change magnification settings. One report described using smart glass to facilitate remote proctoring^17^; however, it seems this particular device was uncomfortable to wear and lacked visual fixation. Another report describes the authors setting up a videoconference call and using laptops to broadcast video feed to the proctor.^18^ The demand certainly exists for a robust system to broadcast procedures from the operating suite and supply the remote proctor with sufficient information on the procedure.

Remote proctoring can be carried out at Houston Methodist as demonstrated by Sachin et al.^19^ At our hybrid suite, a VisitOR1 robot (KarlStorz) is installed, which allows the virtual presence of a proctor to support the operating team by actively participating in the case. The proctor gains access to the multiple video feeds broadcasting from our hybrid operating room via the InTouch Provider software (InTouch Health). Additionally, the proctor can remotely navigate the camera on the robot while bidirectional audiovisual connection contributes to the communication between the on-site team and the remote proctor. This setup proved to be useful in our practice, and a highly reliable alternative for hosting experts as remote proctors.

## Automation of Instrument Counts in the Operating Room

Although retained foreign bodies following surgery are not extensively reported in the literature, their incidence is estimated to be around 1,500 cases per year.^20,21^ Foreign bodies left in the abdomen range from 0.3 to 1.0 in 1,000 cases.^22^ Due to the decline in the number of abdominal surgeries in vascular surgery,^23^ standardization of routine steps is of paramount importance in order to maximize patient safety. The Association of Perioperative Registered Nurses (AORN) recommends instrument count before, during, and after surgery at predefined steps of the operation.^24^ While we do not wish to challenge this practice, additional auxiliary steps could further improve instrument count. As Hendricks et al. demonstrated, radiofrequency-identified (RFID) surgical tools can be used for intraoperative data analytics during cardiovascular surgical procedures.^25^ This system not only prevents inadvertent retained instruments after surgery but also provides data on instrument utilization and selection, which may lead to more refined instrument selection. Combined with the RFID system and the currently used process proposed by the AORN, perioperative errors could be minimized for retained foreign bodies.

## Efficiency Optimization in the Cardiovascular Suite

**Figure 2:**
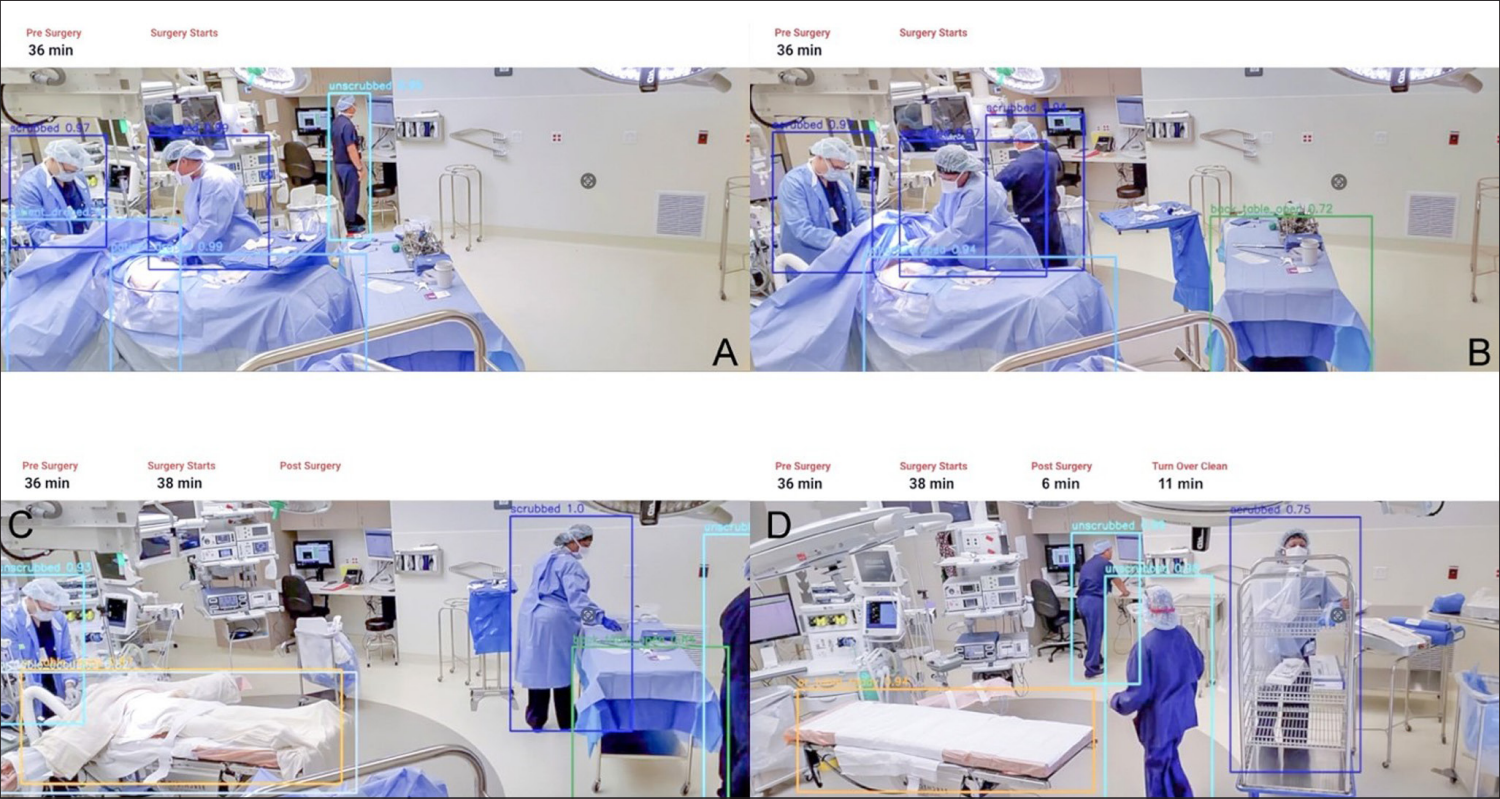
The Apella system in use. The proprietary computer vision technology is capable of identifying and tracking tasks carried out in the operating room from pre-surgery to post-surgery. It identifies **(A)** scrubbed and unscrubbed staff, as well as **(B)** whether the tray is opened on the back table. The system also measures the time needed for pre-surgical tasks, **(C)** how much time it takes to start surgery, and **(D)** how long it takes to finish post-surgical tasks.

Surgery has long surpassed the idealized stereotype of the highly skilled surgeon who individually is able to overcome any challenge. Surgical specialties rely on teamwork more heavily than any other medical profession. The quality of teamwork efficiency may define a procedure as successful or unsuccessful. Apella offers proprietary computer vision technology to passively monitor the operating room and gather data that can be used to improve the efficacy of tasks performed before, during, and after surgery (Figure 2). The captured information is used to provide a highly efficient and streamlined workflow by exploiting the maximum capacity of the operating room and the available resources.

## Monitoring Mental Workload

Mental workload is defined as the tuning between environmental demand and the individual’s capacity.^26^ It can be further classified to perceptual load and cognitive load. Obviously, measuring workload among the medical staff is important, as are further studies to determine cutoff values, where complication rates start to rise exponentially. Such safety measures would further improve patient outcomes. But how can we measure mental workload?

Three distinct approaches are available for monitoring real-time mental workload.^27,28^ Firstly, cardiovascular signals can be measured with electrocardiography. Secondly, brain activity providing information on mental workload can be monitored with electroencephalography and event-related potential. A third option combines eye-movement monitoring with infrared camera, allowing for mental workload assessment in an electrode-free manner. In addition, eye-movement evaluation enables differentiation between perceptual and cognitive workload, thus providing more granular information on mental load.^26^

A systematic review from 2018 identified self-reporting methods (NASA Task Load Index) as the most commonly used tool to assess surgeon’s intraoperative cognitive workload (mostly in an experimental environment, after cadaveric procedures), while electrocardiogram was used most frequently among real-time monitoring approaches.^29^ Self-reporting methods are suitable for the assessment of total cognitive workload following the procedure. In contrast, real-time monitoring techniques provide valuable information on workload fluctuation during the procedure.

Implementation of mental workload assessment in everyday clinical practice promises to be an exciting transition. However, it requires robust research to determine cutoff values for identifying mental overload, which threatens poor outcomes. In addition to improving patient outcomes, mental load evaluation may lead to a decreased burn out rate, if handled appropriately.

## Limitations of Artificial Intelligence in a Hospital Setting

The simpler the clinical question and the collected data, the easier it is to assess certain clinical scenarios using AI. Consequently, the implementation of AI in clinical practice becomes increasingly seamless. However, in more complex applications of AI, such as using neural networks, the process of arriving at a conclusion may be difficult to understand from a “user”/technician standpoint.^30^ Hence, validation of the results generated by AI could become challenging, causing issues with the interpretability of the results.^31^

Since data interpretation is only as good as the data the algorithm already knows, underrepresented patient groups may be at risk of misinterpretation due to selection bias introduced during machine learning.^32^ Finally, the question of accountability of AI systems raises questions regarding responsibility. Naik et al.^33^ suggest that clinicians should be accountable for the output of AI, if they choose to rely on the data; otherwise, they would not be able to justify their actions. The output of AI should be verified and validated in order to build a robust, evidence-based framework around this technology.

## Conclusion

Technological advancements, including AI and telemedicine, have reached and been incorporated into our day-to-day practice. They promise to reshape patient care by guiding us toward an even more efficient and safer workflow, which we strive for every day at Houston Methodist Hospital.

## Key Points

- Technological advances can and should be implemented in the modern healthcare setting specifically in the workflow optimization of the medical staff in order to offer the highest level of patient care.
- The adoption of fall detection-prevention systems and telenursing in everyday clinical practice contributes to improved patient safety and promises to alleviate workload on the nursing staff as well.
- Remote case support with the appropriate equipment eliminates the need for the physical presence of a proctor, allowing for better accessibility to experts from all over the world.
- The use of instrument count automatization systems and computer vision could help to achieve a more streamlined workflow in the operating room.
